# Prefactory comments: promise and enigma of biomarkers for brain injury

**DOI:** 10.3389/fneur.2012.00173

**Published:** 2012-12-06

**Authors:** Andrew I. R. Maas

**Affiliations:** Department of Neurosurgery, Antwerp University Hospital/University of AntwerpEdegem, Belgium

Traumatic brain injury (TBI) represents one of the greatest unmet needs in medicine and public health (Maas et al., 2008). It is a major cause of death and disability and leads to great personal suffering to victims and relatives, as well as huge direct and indirect costs to society (Finkelstein et al., 2006; Faul et al., 2007; Gustavsson et al., 2011). TBI is considered “the most complex disease in our most complex organ.” We now recognize that TBI is not just an acute event but can trigger a chronic process, with progressive injury over hours, days, weeks, months, and even years. The challenges posed by TBI are huge.

Notwithstanding improved understanding of disease mechanisms, appropriate characterization of TBI is complex, and even establishing a reliable diagnosis in subjects with mild injuries, can be challenging. It remains difficult to “look into the brain” and to track disease processes on a continuous basis *in vivo*. Despite the current availability of robust prognostic models, estimates of outcome in individual patients often have a large confidence interval. The emerging field of biomarkers has great potential for improving characterization of TBI: in the acute and subacute phases, biomarkers can aid in the diagnosis of TBI, in tracking disease processes and for establishing more confident prognostic estimates. In the more chronic phases, they may indicate ongoing progressive damage with neuronal and glial cell loss.

This special issue of Frontiers in Neurology on biomarkers in brain injury provides a comprehensive summary of our current knowledge in the emerging field of biomarkers across the TBI spectrum from initial injury to long term outcome. Despite the extremely promising results presented, this issue also illustrates some of the gaps in our knowledge, thus stimulating further research and development. Why, for example, is it so much more difficult to find reliable serum biomarkers for brain injury than for, for example, myocardial disease (e.g., troponin)? Could this be perhaps that we still have insufficient knowledge of how degradation products from brain tissue are removed into the venous blood? Is this directly into the venous system, or indirectly by flow of the extracellular fluid draining via the cerebrospinal fluid into the sagittal sinus? Basic understanding of such mechanisms would be of great relevance toward optimal biomarker sampling. Whilst many studies on the prognostic value of biomarkers show clear prognostic effects, it should be realized that numbers in these studies in general are small and that the added value of biomarkers as prognostic indicators over and above other predictors has not yet been adequately shown in multivariable analyses. The concept of being able to differentiate between neuronal and glial injury based upon biomarkers is exciting. This topical issue will also address relations between laboratory markers and other biomarkers such as imaging modalities. By definition characterization and classification of brain injury is multidimensional. Better characterization with the aid of biomarkers can be expected to facilitate Precision Medicine, a concept recently advocated by the US National Academy of Science (2011). Precision Medicine aims for appropriate targeting of management and individualizing treatment approaches based upon more precise characterization of the disease process. Achieving these goals and establishing the role of biomarkers herein will require confirmation of promising results from proof of concept studies in larger numbers. This can best be accomplished in multidisciplinary, international collaborations, collecting high quality prospective data in observational studies in parallel to continued basic science research.
